# The Position of the Territorial Medical Officer

**Published:** 1916-11-11

**Authors:** 


### THE POSITION OF THE TERRITORIAL MEDICAL OFFICER.

With the prolongation of the Great War and the steady drain upon the medical profession to supply the medical needs of our now gigantic armies in the field, a question is coming into prominence which has hitherto aroused but little attention: that is, the position of the officers of the Territorial (and Special Reserve) Medical Services, as compared with that of the temporarily commissioned. Briefly, medical officers of the Territorial and Special Reserve branches of the Royal Army Medical Corps were mobilised for service immediately on the outbreak of war, and have no prospect whatever of returning to civilian life, unless they are invalided through wounds or sickness. The temporarily commissioned, on the other hand, sign an agreement for twelve months' service only, with the option at the end of that term of service to re-sign or to return to civil life. Even when, as is usually the case, the temporarily commissioned sign for a further twelve months in the Army, they can and not infrequently do take a holiday of a few weeks or months between the two tours of service, a not inconsiderable advantage which is denied to the Territorials and Special Reserve.

This difference in treatment, now that compulsory service for all is the law of the land, is very unfair, though it may be, and to some extent doubtless is, unavoidable. In effect it punishes a man, often very heavily, for having been such a fool as to take trouble and spend money in peace-time preparing himself to meet the very emergency which has overtaken the country. Many and many a Territorial officer of the R.A.M.C. has been giving up a fortnight of his time to his country every year, and been out of pocket into the bargain, only to find when war comes that he is half ruined as a reward for his patriotism; whereas his neighbour, who has never done anything for the State, gets off with a period of twelve months' service or, if he be over forty-one, with none at all. Small wonder that the Territorial officers feel sore about the matter. They may be forty-one, fifty-one, or sixty-one, yet are not regarded as too old for the Army; and they do not see why their rivals or colleagues who happen to be turned forty-one should be left at home undisturbed, to benefit by the absence of the more patriotic men from their practices. The anomaly is indefensible; though it may not be easy just now to point out a remedy, for the essential injustice is part and parcel of our unfair and ridiculous military system. There is, however, an impression about that the Central Medical War Committee is hard put to keep Sir Alfred Keogh supplied with the men he wants; and rumours are heard that the age limit for compulsory service amongst doctors may be raised. This cannot be done without the consent of Parliament, and it is very doubtful whether Parliament would agree to treat any section of the population on a different footing from the rest. Meanwhile the Territorial R.A.M.C. officers have entered the third year of absence from their practices; and many of them after the war will practically have to begin the world again, a serious matter for the middle-aged and no light one even for the younger men.

